# Safety and efficacy of one-hole split endoscope technique for surgical treatment of thoracic ossification of the ligamentum flavum

**DOI:** 10.1038/s41598-024-55055-z

**Published:** 2024-02-22

**Authors:** Qi Sha, Zhengdong Huang, Jinhao Liu, Peng Ge, Yong Zhang, En Song, Zhaozhong Sun, Tenyue Zhu, Cailiang Shen, Jun Qian

**Affiliations:** 1 Department of Orthopedics, The First Affiliated Hospital of Anhui Medical University, Hefei, 230032 Anhui China; 2 Department of Sports Medicine, The First Affiliated Hospital of Kunming Medical University, Kunming, 650032 Yunnan China; 3 Department of Spine, Binzhou Medical University Hospital, No. 661 Huanghe 2nd Road, Binzhou, Shandong China; 4 Department of Orthopaedics, The Sixth Medical Center of PLA General Hospital, Beijing, 100048 China

**Keywords:** Spinal cord diseases, Spine structure

## Abstract

Surgical intervention is typically recommended for thoracic ossification of the ligamentum flavum (TOLF). This study aimed to evaluate the efficacy and safety of a novel non-coaxial one-hole split endoscope (OSE) technique for treating TOLF. We performed OSE procedure on 13 patients with TOLF from June 2022 to July 2023. The mean operative time was 117.5 ± 15.4 min. VAS scores for lower limbs decreased from 6.5 ± 0.8 preoperative to 1.6 ± 0.4 at the last follow-up (P < 0.001). ODI scores improved from 62.4 ± 5.7 preoperative to 18.6 ± 2.2 at the last follow-up (P < 0.001), and mJOA scores increased from 5.1 ± 1.6 preoperative to 8.4 ± 1.5 at the latest follow-up (P < 0.001). All patients achieved ASIA scale grade D or E at the final follow-up, except for two patients remained residual limb numbness. None of the thirteen patients suffered from severe perioperative complications. The OSE technique proves to be a safe and effective procedure for treating TOLF or even with dura mater ossification, characterized by minimal surgical trauma, relatively smooth learning curve and flexible operation.

## Introduction

Thoracic ossification of the ligamentum flavum (TOLF) is a major cause of thoracic spinal stenosis, significantly impacting patients' quality of life and even leading to paraplegia in severe cases^1,2^. Ectopic ossification replaces the ligamentum flavum^3^, with a high incidence in the lower thoracic vertebrae in Asian countries^4,5^. Due to the disease's continuous progression and irreversibility, surgical decompression is necessary for patients with neurological symptoms^6^. Traditional posterior laminectomy not only reduces spinal stability but also increases the incidence of complications, including kyphosis, surgical trauma and chronic back pain^7,8^.

Recent advancements in minimally invasive spine techniques have shown the prospective application of full endoscopic spine surgeries (FESS) in treating TOLF^9–11^. However, these procedures are limited because of the steep learning curves in FESS, and controversies remain regarding approach selection, surgical instruments, and decompression strategies. Unilateral biportal endoscope (UBE) approaches have also shown good outcomes in treating TOLF^12,13^. However, two incisions should be made on the skin to make surgical window in UBE technique, and this may lead to more surgical trauma. OSE technique was first proposed and applied in clinical in 2019^14^. Compared with UBE approach, just one incision approximately 1.5–2 cm on the skin is made in OSE technique. Furthermore, the endoscope and surgical instruments are splited but both locate in the same incision through OSE approach. It not only minimizes tissue damage but also increases flexibility in surgical procedures by placing the endoscope and surgical instruments in the same small incision. Our group has successfully applied OSE procedure to treat various spine disease including TOLF.

This study aims to introduce the novel OSE technique for removing thoracic ossification of the ligamentum flavum and to evaluate its safety and efficacy for minimally invasive surgical treatment of TOLF.

## Materials and methods

All patients provided written informed consent, and the procedures were approved by the Ethics Committee of hospital (2022WKZ No. 33). Furthermore, our study meets the guidelines of the Anhui Institute of science and technology.

### Patients

In the present study, 13 patients with TOLF underwent OSE surgery were analyzed prospectively. Surgeons with over 10 years of experience in endoscopic thoracic spinal surgery conducted all procedures. There were eight women and five men, with an average age of 60.5 ± 10.1 years (range, 40–77). All patients reported severe back or leg pain, numbness, and weakness in the lower extremities, which worsened gradually over a period of 3 to 12 months (average 6.8 months). Physical examinations and MRI were performed to demonstrate neurological deficits resulted from the dorsal side compression of spinal cord. 3D-CT showed that the cause of spinal cord compression as a result of TOLF, and 14 ossifications of the ligamentum flavum on 13 patients, including ossification of the dura mater in one patient. The inclusion criteria were as follows: (1) walking instability, numbness and weakness of lower extremities, sphincter dysfunction, and ineffective conservative treatment, (2) single or two segment stenosis with ossification of the ligamentum flavum, and (3) clinical symptoms consistent with imaging findings. The exclusion criteria were as follows: (1) previous surgical segment fractures, infections, or tumors, (2) related segment with spinal deformities, instability, and surgical history, (3) intermittent claudication with lower limb vascular factors, and (4) general contraindications of anesthesia or coagulation dysfunction.

### Surgical procedures

**Figure 1:**
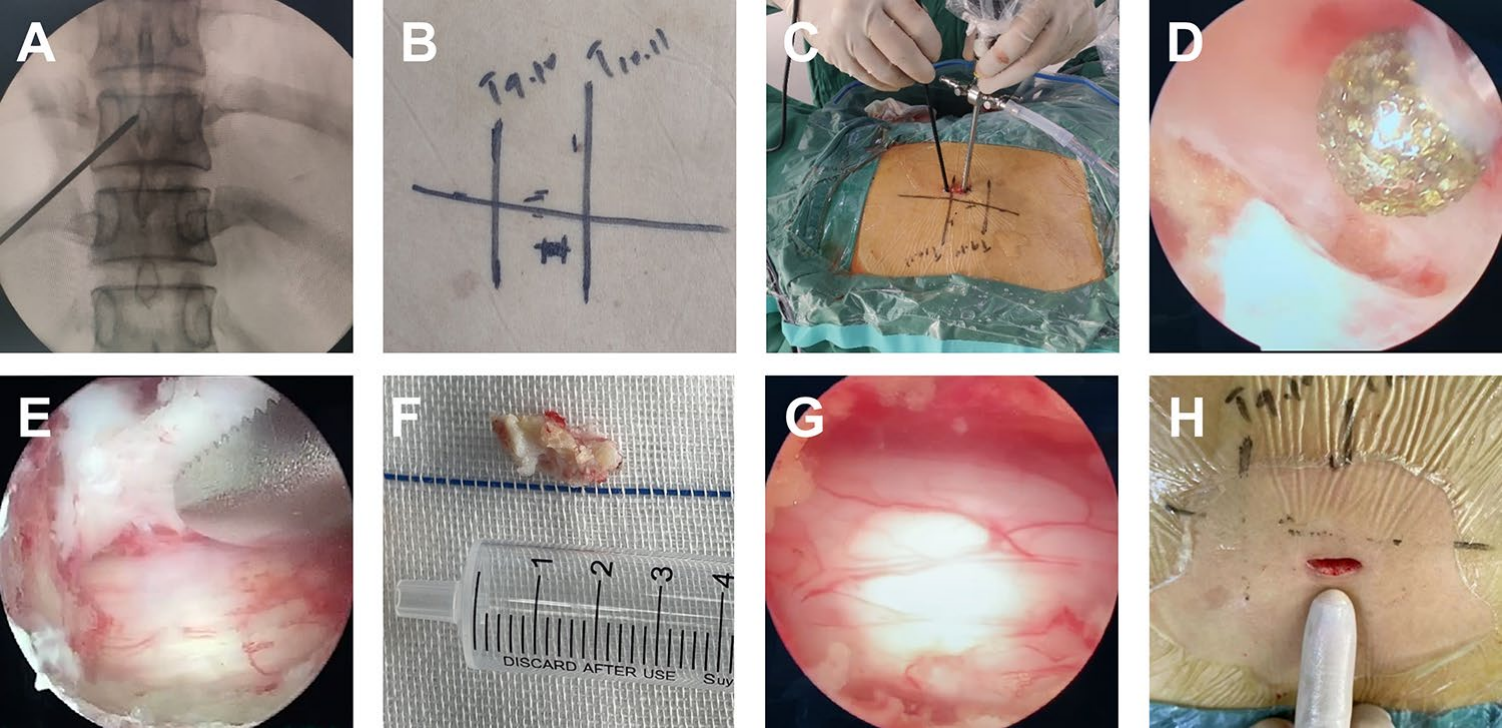
Workflow of OSE technique performed in patients with TOLF. **(A,B)** Confirmation of target segment and marking incision on the skin. **(C)** Endoscope and surgical instruments split but locate in the same incision. **(D)** Using microscopic drill to remove the ossification lesion layer by layer. **(E)** Using nucleus pulposus forceps to remove the ossification in one piece. **(F)** A piece of ossification lesion. **(G)** Complete decompression of spinal cord with good dural pulsation. **(H)** Minimally invasive incision approximately 1.5–2 cm.

The surgical procedures were conducted in a standard operating room with the patient in a prone position. X-ray fluoroscopy was used to confirm the target intervertebral space and the location of the incision (Fig. 1A). After routine disinfection, the side with the most severe imaging or symptoms was chosen as the surgical approach side. A longitudinal incision of approximately 1.5–2 cm was made at the inner lower edge of the upper pedicle and the transition of the vertebral lamina towards the articular process (Fig. 1B). A dilator was used to expose the surface of the vertebral lamina. The endoscope and surgical instruments were placed into the same incision (Fig. 1C), and a surgical window was created using a continuous saline perfusion system. The space for lamina (intervertebral) fenestration and decompression was selected based on the location of the ossification lesion. A microscopic drill was used to grind the lamina and ossified ligamentum flavum layer by layer (Fig. 1D). If necessary, the nerve dissector and miniature nucleus pulposus forceps were used to separate and remove the ossification lesion in piece (Fig. 1E). After bilateral decompression of the spinal cord was completed, a good appearance or pulsation of the dural sac could be seen under the endoscope (Fig. 1F). Electrophysiological monitoring and intraoperative arousal techniques were applied to detect immediate neurological function. Intraoperative hemostasis was performed, and the incision was sutured without drainage tubes (Fig. 1G, H). The workflow of OSE technique performed in patients with TOLF is shown in Fig. 1.

### Post-operative treatment

Neurotrophic drugs were provided to each patient, and rehabilitation of both lower limbs was encouraged as soon as possible after OSE surgery. All patients underwent 3D-CT and MRI on the first day after surgery.

### Follow-up

The follow-up consisted of questionnaires or personal contact with patients (either by phone, outpatient review or clinical investigation). Radiological examinations were used to evaluate the patient’s condition and recurrence of TOLF when necessary.

### Clinical outcome measurements

To evaluate the efficacy and safety of OSE technique for minimally invasive surgical treatment of TOLF, operative time, complications, levels of erythrocyte and hemoglobin before and after surgery were recorded. Postoperative 3D-CT and MRI of spine were used to identify the efficacy of surgical decompression by OSE procedure. VAS score, ODI score, modified JOA^15^, ASIA impairment scale and MacNab criteria were also used to assess the clinical outcomes.

### Statistical analysis

All data were analyzed using SPSS Version 26.0 software. Continuous data were presented as the mean ± standard deviation, and statistical tests were performed to compare preoperative and postoperative data. A significance level of P < 0.05 was considered statistically significant.

## Results

**Figure 2:**
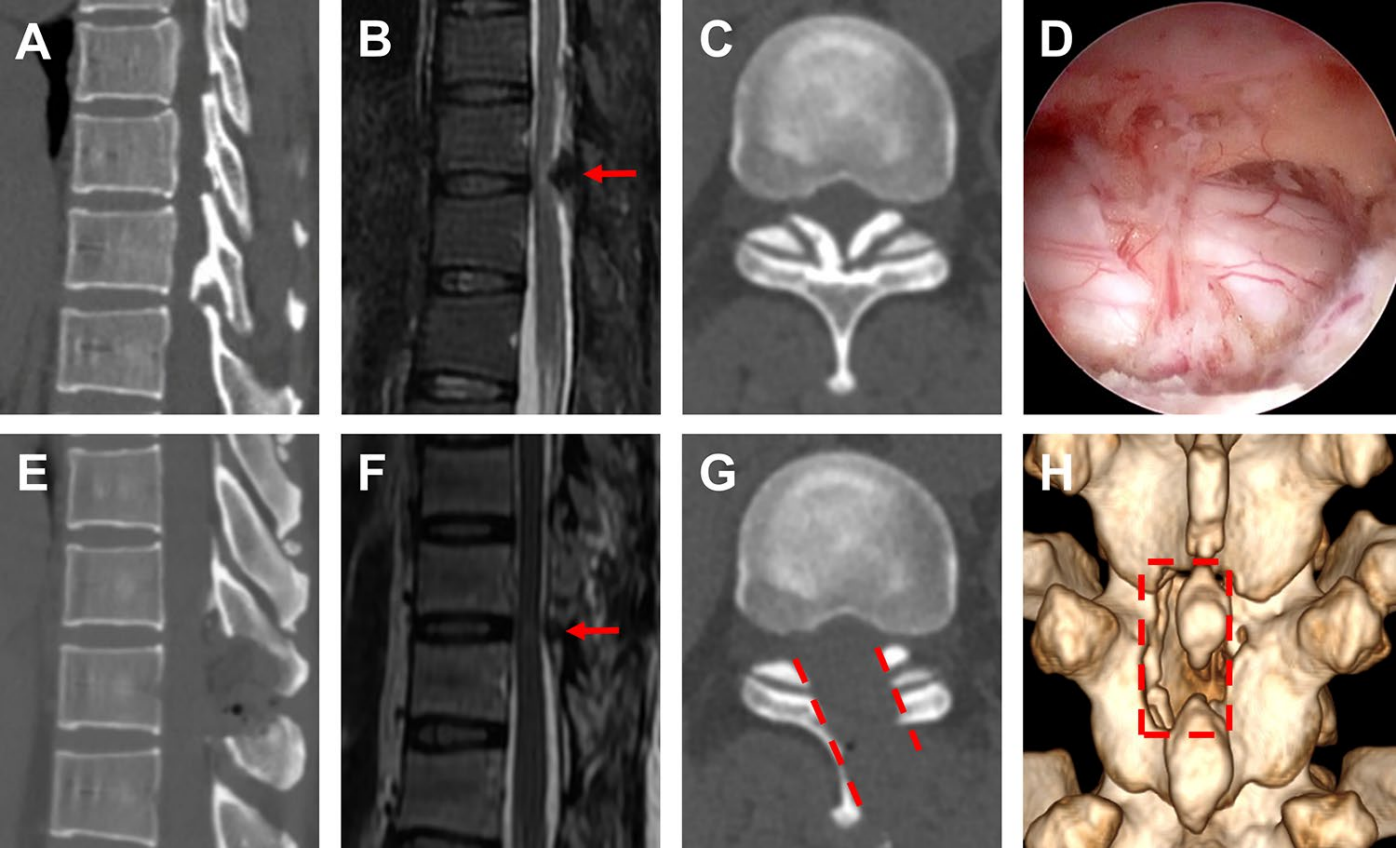
Single segment TOLF treated with OSE procedure. **(A–C)** Preoperative sagittal, axial CT and MRI images of TOLF, and the ossification lesion compressed the spinal cord at T10/11 (As shown by the red arrow). **(D)** The view of decompression of spinal cord during operation on endoscope. **(E–G)** Postoperative sagittal, axial CT and MRI images, demonstrated a complete remove of ossification lesion (As shown by the red arrow and dashed). **(H)** Postoperative 3D-CT image showed the range of unilateral laminectomy at T10 and T11 (As shown in the red dashed region), bilateral decompression of spinal cord through unilateral approach.

**Figure 3:**
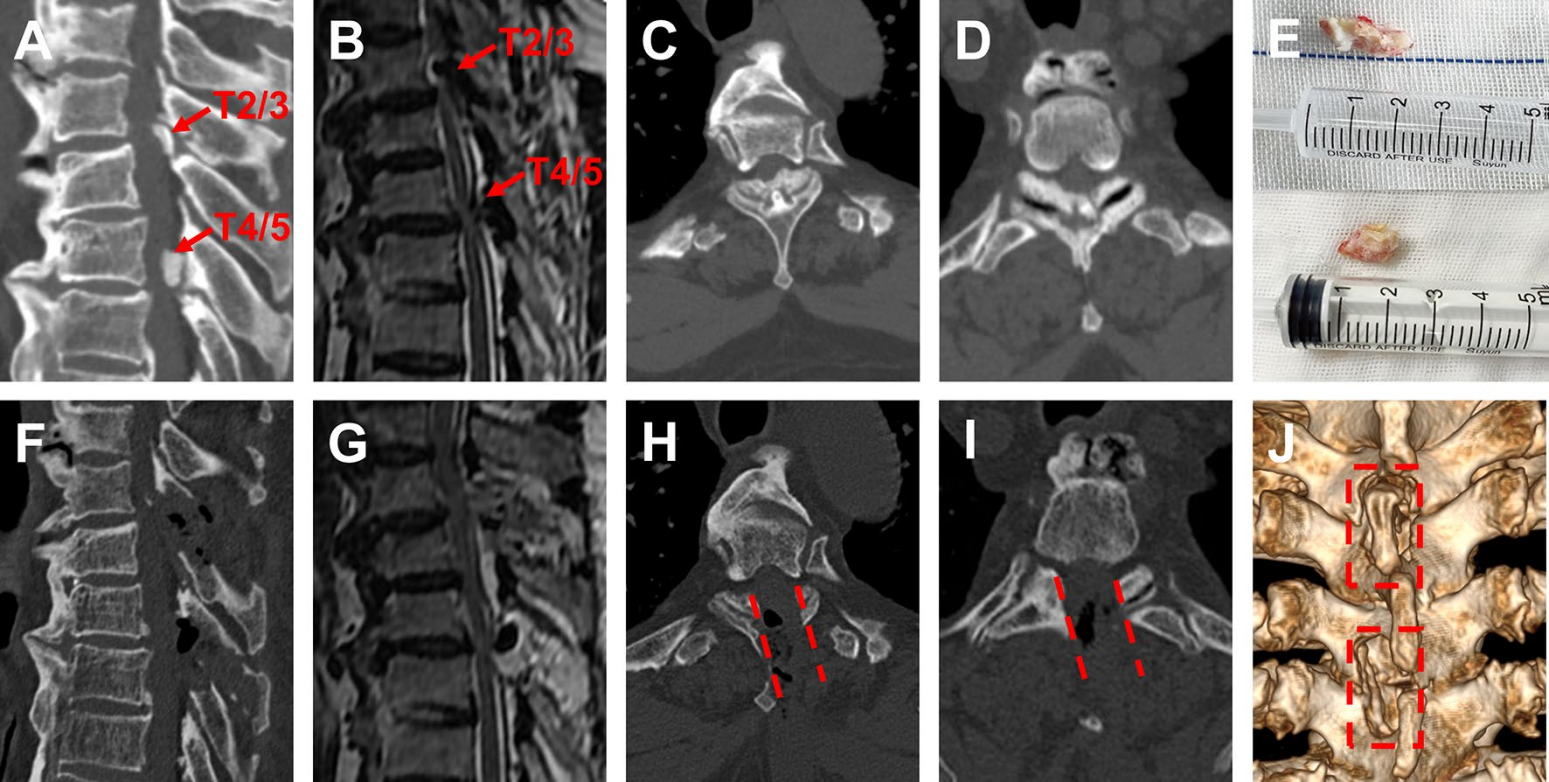
Two segments TOLF treated with OSE procedure. **(A–D)** Preoperative sagittal, axial CT and MRI images of TOLF, and the ossification lesion compressed the spinal cord at T2/3 and T4/5. Long T2 signal in spinal cord on MRI (As shown by the red arrow). **(E)** Remove the ossification lesions of T2/3 and T4/5 in piece. **(F–I)** Postoperative sagittal, axial CT and MRI images, demonstrated a complete remove of ossification lesion (As shown by the red dashed) and good decompression of spinal cord at T2/3 and T4/5. **(J)** Postoperative 3D-CT image showed the range of unilateral laminectomy at T2/3 and T4/5 (as shown in the red dashed region), bilateral decompression of spinal cord through unilateral approach.

**Figure 4:**
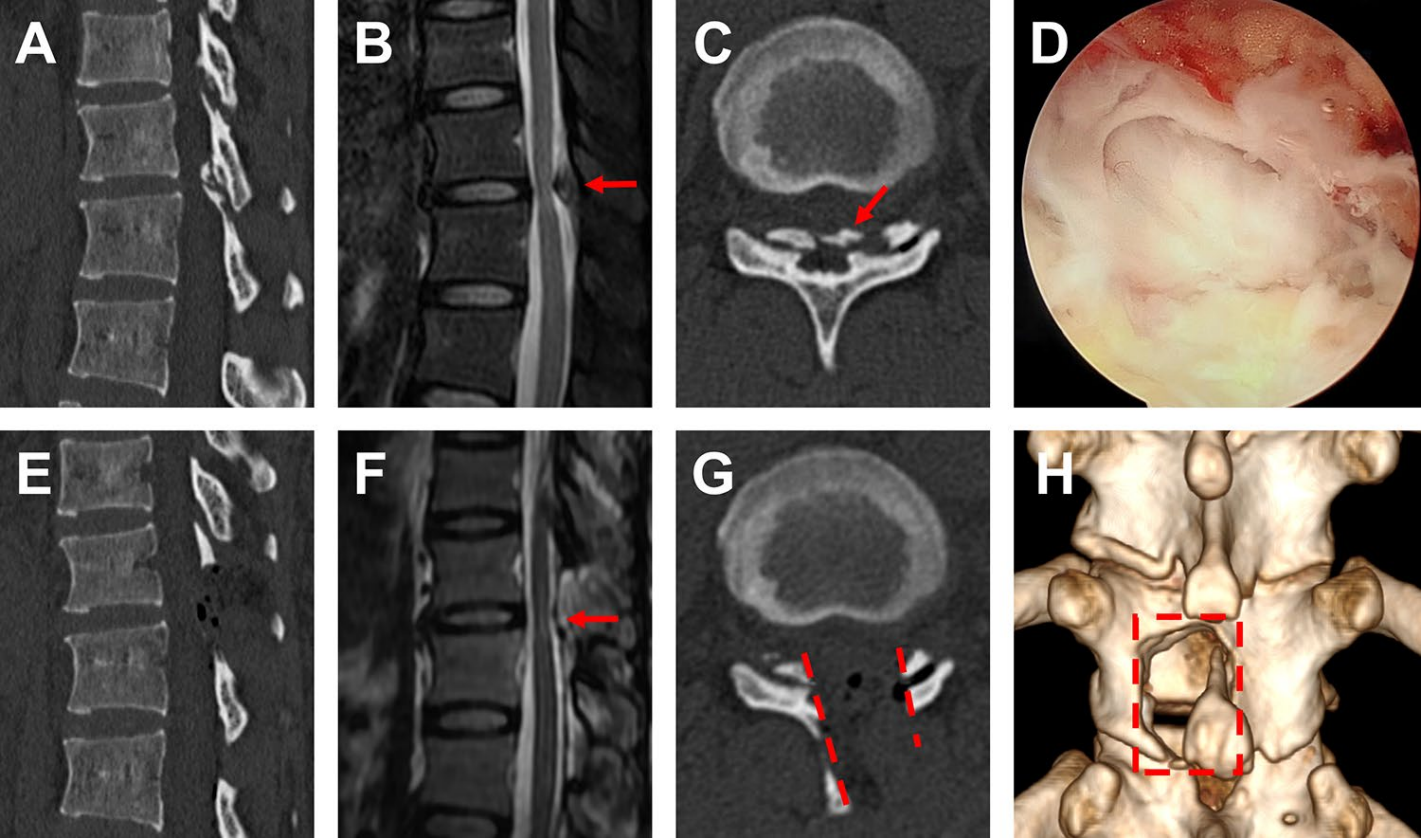
DO with TOLF treated with OSE procedure. **(A–C)** Preoperative sagittal, axial CT and MRI images of TOLF, and the ossification of dura mater compressed the spinal cord at T11/12 (As shown by the red arrow). **(D)** The view of spinal cord during operation after removing the ossification of dura mater on endoscope. **(E–G)** Postoperative sagittal, axial CT and MRI images, demonstrated a complete remove of ossification lesion originating from dura mater and ligamentum flavum (As shown by the red arrow and dashed). **(H)** Postoperative 3D-CT image showed the range of unilateral laminectomy at T11/12 (As shown in the red dashed region), bilateral decompression of spinal cord through unilateral approach.

OSE procedure was successfully performed in all patients, with complete removal of ossification and restoration of spinal cord morphology confirmed by postoperative 3D-CT and MRI (Figs. 2, 3 and 4). The mean procedure time was 117.5 ± 15.4 min (range 96–147). Because of the continuous saline perfusion system used during operation, intraoperative blood loss is difficult to calculated accurately, but the mean pre-operative hemoglobin value was 132.4 ± 19.8 g/L in comparison to 128.6 ± 21.7 g/L after surgery, which did not present a significant difference (P = 0.284). Pain of lower limbs was relieved significantly in all patients on the first day after surgery. All patients underwent a follow-up period of at least 3 to 15 months. The mean VAS score of lower limb was 6.5 ± 0.8 before surgery and dropped to 1.6 ± 0.4 at the last follow-up, which represented a statistically significant difference (P < 0.001). Similarly, the mean ODI score decreased from 62.4 ± 5.7 preoperatively to 18.6 ± 2.2 at the last follow-up (P < 0.001). The modified JOA increased from 5.1 ± 1.6 before surgery to 8.4 ± 1.5 at the last follow-up (P < 0.001) (Table 1).

**Table 1:** Comparison of preoperative and postoperative clinical outcomes.

Variable	Preoperative	1 month postoperative	Final follow-up
VAS for leg pain	6.5 ± 0.8	2.9 ± 0.8	1.6 ± 0.4
ODI score (%)	62.4 ± 5.7	33.6 ± 5.6	18.6 ± 2.2
JOA score	5.1 ± 1.6	6.5 ± 1.6	8.4 ± 1.5
ASIA impairment scale	C (5)	C(2)/D(3)	D(2)/E(3)
	D (8)	D(2)/E(6)	E(8)
MacNab criteria	Excellent (4), good (7), fair (2), poor (0)		

Values are presented as numbers or mean ± standard deviation.

*VAS* visual analogue scale, *ODI* Oswestry Disability Index, *JOA* Japanese Orthopaedic Association.

According to ASIA impairment scale, 5 patients were rated as C grade before surgery and increased to grade D in 2 patients and E in 3 patients at the final follow-up. The remaining 8 patients were classified as grade D before surgery, and all improved to grade E at the last follow-up. According to the MacNab criteria, it showed satisfaction with the surgery (4 excellent, 7 good, 2 fair) (Table 1). Weakness of lower extremities improved significantly, pain disappeared completely in all patients and residual limb numbness occurred in 2 patients until the last follow-up. In addition, none of 13 patients suffered from complications such as incision hematoma or infection, cerebrospinal fluid leakage and spinal cord injury. The incision was less than 2 cm in each patient, and there was no additional surgical damage of muscle and organ except for blunt separation to make the surgical window.

## Discussion

TOLF is a relatively rare yet perilous condition associated with thoracic spondylotic myelopathy, which predominantly occurs in the lower thoracic segment^16^, with a reported prevalence range from 3.8 to 63.9%^3,6,17^. In line with prior studies, our investigation revealed that 8 out of 13 cases (61.5%) manifested below the T9 segment. This occurrence may be attributed to factors such as spinal mobility and mechanical stress^5,18^. While there remains no universal consensus on the treatment principles of TOLF, existing studies strongly suggest that conservative therapy is inadequate in averting spinal cord compression-related complications. Symptomatic patients are advised to undergo early surgical intervention to achieve superior clinical outcomes^6,19^.

Traditional open surgical procedures, such as posterior laminectomy or decompression and fusion, heighten the risk of intraoperative trauma and perioperative complications, impeding swift functional recovery^7^. A comprehensive retrospective study conducted by N. S. Osman et al.^6^ revealed that the incidence of postoperative complications following laminectomy stood at 35%. Dural tears occurred in a striking 18.4% of cases, cerebrospinal fluid leakage in 12.1%, infections in 5.8%, and early neurological deterioration in 5.7%. Simultaneously, these procedures can disrupt bone structures and the posterior ligament complex, potentially leading to spinal instability and degeneration of adjacent segments^8,20^. With the evolution of endoscopic surgical tools, endoscopic techniques in spine surgery have gradually found utility in the treatment of TOLF. When compared with open surgeries, FESS procedures offer benefits in terms of minimal invasiveness and expedited recovery^9–11^. Nevertheless, FESS approaches have shown some new issues including limitations due to the coaxial approach in a single hole endoscope, a steep learning curve, and necessitating surgeons to possess extensive experience in endoscopic surgery. The UBE technique has also been described as a surgical method for addressing thoracic spondylotic myelopathy resulting from ossification of the ligamentum flavum^12,13^. Differing from FESS technique, the UBE technique deploys the endoscope and surgical instruments through two separate incisions, providing a larger operating space and partly reducing operational complexity.

To further reduce surgical trauma, a novel minimally invasive spine technique named OSE technique was first designed and applied by one of authors in present study in China in 2019^14^. In contrast to the UBE technique's dual incisions, the OSE technique utilizes a single incision, housing both the endoscope and the surgical instruments. This configuration minimizes soft tissue damage. Overall, FESS, UBE, and OSE techniques all have the characteristic of minimally invasive, good retention of spinal stability and significantly reducing surgical trauma compared to traditional open surgery. However, the FESS technique, because the endoscope and instruments are confined to the one fixed channel, may suffer from a steep learning curve in practice. The UBE technique separates the endoscope from the working channel through two small incisions on one side, eliminating coaxial limitation and resulting in a relatively smooth learning curve. The OSE technique, as a further innovation based on the FESS and UBE techniques, the working channel and the observation channel are separated but located in the same incision, which greatly reduces the operating difficulty. OSE technique has a smoother learning curve, and realizes minimally invasive surgical operation only with a single small incision. For single segment lesions, OSE and FESS techniques can achieve surgical decompression through a single small incision, further reducing tissue damage compared to UBE approach. Compared with FESS technique, OSE and UBE approach both provide more flexibility in surgical operations with a variety of instruments and a relatively smoother learning curve.

In this study, 13 patients with TOLF underwent OSE surgery to remove the ossification of ligament flavum and dura mater. Sato et al.^21^ categorized the CT imaging manifestations of TOLF into five types. For segmental ossification such as lateral type, extended type and enlarged type, it is necessary to find the junction between the normal and ossified ligamentum flavum and then gradually remove ossification layer by layer with the micro-grinding drill to expose the dural sac by OSE procedure (Figs. 1D and 2). When the ossification is the fused or tuberous type, a longitudinal groove could be created alone both sides of the ossification, allowing for comprehensive decompression and removal of hypertrophic ligaments and ossifications in piece (Fig. 3). Dural ossification (DO) is often presented as "tram track sign" on CT, it reported that nearly 60% of fusion or tuberous-type ossifications are combined with DO^22^. In cases of DO, a microscopic drill may be used to reduce the ossification to an eggshell shape, after which it can be delicately removed using a nerve dissector and micro-nucleus pulposus forceps. In our study, we ascertained that the OSE technique is adept at effectively mitigating spinal cord compression in TOLF patients with DO (Fig. 4). Despite the endoscope and operation instruments both in a small incision during OSE procedure, OSE technique can significantly reduce the difficulty and danger to perform bilateral spinal cord decompression through unilateral approach in patient with TOLF and even DO as a result of non-coaxial working channel. In this paper, all patients showed significant improvement in lower extremities and leg pain after OSE surgery. Notably, none of 13 patients suffered from cerebrospinal fluid leakage, spinal cord injury or incision infection. OSE procedure provides several advantages : (1) significantly reduced surgical trauma and perioperative complications, (2) operating flexibility due to non-coaxial working channel splitting the endoscope and surgical instruments, (3) relatively smooth learning curve, and (4) less intraoperative radiation damage.

In the present study, all patients retained mobility after OSE surgery. The symptoms of pain and weakness of lower extremities were significantly improved until the last follow-up. Only two cases remain residual limb numbness and no patients had surgical related complications. As far as we know, this is the first study to establish the safety and efficacy of the OSE technique for treating TOLF and even DO.

This study has several limitations. First, the sample size of patients with TOLF underwent OSE surgeries was relatively small. Furthermore, we did not systematically compare the differences in learning curves between OSE, UBE and FESS technique. Therefore, large-scale, prospective study is needed in the future. The improvement in JOA score in this study was relatively mild, which may be related to severity of symptoms, preoperative ASIA grade and shorter follow-up time. While we have observed positive early clinical outcomes, we acknowledge that this procedure warrants further refinement and long-term follow-up.

## Conclusion

Our study suggests that the OSE surgery is a safe and effective technique for minimally invasive surgical treatment of TOLF and even DO. It offers minimal surgical trauma, a relatively smooth learning curve and non-coaxial limitations during surgery. The OSE technique provides a new and promising minimally invasive surgical strategy for treating TOLF.

## Data Availability

The data from this study can be available from the corresponding author on reasonable request.
